# Subgingival Delivery of Statins as an Adjunct in the Non-Surgical Treatment of Periodontitis: A Systematic Review

**DOI:** 10.3390/biomedicines13010182

**Published:** 2025-01-13

**Authors:** Magdalena Maria Pietrzko, Maciej Pietrzko, Wojciech Niemczyk, Dariusz Skaba, Rafał Wiench

**Affiliations:** 1 Pietrzko Stomatology, Budowlanych 1 Street, 43-300 Bielsko-Biała, Poland; pietrzko@gmail.com; 2 Department of Periodontal Diseases and Oral Mucosa Diseases, Faculty of Medical Sciences in Zabrze, Medical University of Silesia, Pl. Traugutta 2, 41-800 Zabrze, Poland; niemczykwojciech00@gmail.com (W.N.); dskaba@sum.edu.pl (D.S.); rwiench@sum.edu.pl (R.W.)

**Keywords:** periodontal disease, diabetes, smoking, clinical parameters, simvastatin, rosuvastatin, atorvastatin calcium

## Abstract

Background/Objectives: The gold standard in the non-surgical treatment of periodontitis is scaling and root planning (SRP). In recent years, studies have emerged suggesting additional clinical benefits from the use of statins as an adjunct to classical periodontal disease treatment. The aim of the present study was to review the relevant literature relating to the subgingival use of statins as an adjunctive treatment to the classical, non-surgical treatment of periodontitis, with a particular focus on groups with general factors that may affect the outcome of treatment. Methods: The authors conducted a systematic review following the PRISMA 2020 guidelines. The electronic literature search conducted included the MEDLINE (PubMed) database, Web of Science, Scopus, and Google Scholar from 1 January 2012 to 14 June 2024. The keywords used for the PubMed search were determined with the help of the MeSH Browser Tool and were as follows: Periodontitis [Mesh] AND Statin [Mesh] OR Simvastatin [Mesh] OR Atorvastatin [Mesh] or Rosuvastatin Calcium [Mesh]. Based on the authors’ inclusion and exclusion criteria, 20 results were included in the review, out of 937. Results: The improvement was more pronounced in patients without systematic diseases compared to those with type II diabetes and in non-smokers compared to smoking patients. Greater improvements in clinical and radiological parameters were seen in patients diagnosed with aggressive periodontitis compared to patients with chronic periodontitis. Conclusions: This literature review led the authors to the conclusion that statins applied locally might be competent agents for improving the therapeutic outcomes of SRP.

## 1. Introduction

The progressive destruction of the tooth-supporting structure is a hallmark of periodontitis, a chronic, multifactorial inflammatory disease linked to a dysbiotic biofilm that can result in tooth loss. While bleeding and swelling may occur as the disease progresses, periodontitis is typically regarded as a silent condition [1,2]. According to the most recent classification, there are four stages of periodontitis, numbered I through IV, and are graded A through C. Conventional procedures such as scaling and root planning (SRP) and surgical periodontal treatment (SPT) are used to control infection, reduce probing pocket depth (PPD), and improve clinical attachment level (CAL) [3]. Since a minimally invasive approach with proactive procedures is currently preferred, researchers highlight the potential benefits of topical statins as an adjunct to non-surgical treatment.

Statins are a type of drug that was introduced in 1987 [4]. They are molecules of fungal origin [5], and their primary functions are to lower cholesterol levels by specifically blocking 3-hydroxy-3-methylglutaryl coenzyme A (HMG-CoA) reductase, an enzyme that limits the rate at which cholesterol is synthesized [6]. They have provided outstanding contributions to the prevention of cardiovascular disease. Statins have the ability to lower cholesterol levels and express dynamic functions. Several pleiotropic effects, such as anti-inflammatory modulation of vascular response, microvascular reperfusion, antimicrobial effect, and enhancement of wound healing processes, are caused by statins’ reduction in mevalonate pathways [7]. It appears that some of their anti-inflammatory qualities, such as the inhibition of MMP-9 and TNF-alpha, contribute to their beneficial cardiovascular effect [8,9]. It is worth highlighting that the levels of MMP-9 and TNF-alpha seem to play a role in the tissue damage caused by chronic periodontitis [10,11]. Statins have been shown to inhibit osteoclast differentiation and improve the production of bone anabolic factors, such as vascular endothelial growth factor (VEGF) and bone morphogenetic protein 2. These factors contribute to osteoblast differentiation and bone formation [12].

Statins also have immunomodulatory, antioxidant, and antithrombotic actions, as well as help inhibit tumor growth and metastasis [13]. They have direct anti-inflammatory and plaque-stabilizing effects through the inhibition of monocyte recruitment and adhesion to the endothelium, as well as the improvement of endothelial function [14]. There are many studies confirming the pleiotropic effects of statins at the molecular level [15].

There is no successful treatment without side effects, just like with any other medication. The most common adverse effects to systemic use of statins consist of gastrointestinal distress, liver and muscle toxicity, and interactions with other medications that the individual may be taking. Statins, however, have good safety and effectiveness records as well as a profile where the frequency of these negative effects is low [16].

There are several types of statins, such as atorvastatin, ezetimibe (usually administered in combination with another drug), fluvastatin, lovastatin, pitavastatin, pravastatin, rosuvastatin, and simvastatin [17]. The application of statins in the treatment of periodontitis has been evaluated via local application in the periodontal pockets. Local application of simvastatin, rosuvastatin, or atorvastatin under preclinical conditions resulted in a reduction in bone resorption, the levels of inflammatory markers, inflammatory infiltration, while causing an increase in anti-inflammatory mediators and antioxidant substances [18,19].

The aim of the present study was to review the relevant literature relating to the subgingival use of statins as an adjunctive treatment to the classical, non-surgical treatment of periodontitis, with a particular focus on groups with general factors that may affect the outcome of treatment.

## 2. Materials and Methods

### 2.1. Focused Question

A systematic review was conducted following the PICO framework [20] as follows: in patients with periodontitis (population), does the adjunctive use of statins applied locally in conjunction with SRP (intervention) compared to SRP alone (comparison) result in a more effective improvement of clinical parameters (outcome)?

### 2.2. Search Strategy

The systematic review was registered in the PROSPERO database on 26 June 2024, under the ID number CRD42024557129. The protocol of this systematic review can be accessed there. This review was conducted following the Preferred Reporting Items for Systematic Reviews and Meta-Analyses (PRISMA 2020) guidelines [21]. The electronic literature search included the MEDLINE (PubMed) database, Web of Science, Scopus, and Google Scholar from 1 January 2012 to 14 June 2024. The rationale for this period was twofold. Firstly, the authors aimed to conduct a comprehensive review of contemporary research in this field. Additionally, there was a significant increase in the number of publications on this topic since 2012. Table 1 presents the syntaxes utilized, the limitations imposed, and the number of results obtained for each database searched.

The authors also conducted a “snowball” search to find more studies. This involved looking through the reference lists of publications which were then chosen for a full-text review. We also used Google Scholar to find and confirm other studies that had cited the chosen publications. Another requirement was that the electronic search was limited to studies in English. To reduce the risk of bias when searching for an article, the authors decided to not limit the search to randomized control trials. This decision was made because the labeling of papers is not always accurate, and the most recent papers may not have been labeled yet.

Two authors (M.M.P. and M.P) searched the databases independently using the same search terms. Each author evaluated whether the study in question satisfied all the inclusion requirements after searching and choosing potential studies for this review. The two authors jointly searched the literature to find the needed data in order to gather the data from the included studies.

### 2.3. Selection of Studies

The objective of this systematic review was to assess the efficacy of the use of subgingival statins in combination with the SRP procedure compared to SRP alone or with a placebo gel. The hypothesis was that statins applied locally would facilitate a greater improvement in clinical parameters in the context of non-surgical periodontal therapy. The criteria for the inclusion of articles and the exclusion of articles from this review are presented in Table 2.

### 2.4. Risk of Bias in Individual Studies

During the first stage of the study selection process, each reviewer evaluated titles and abstracts independently to minimize potential biases. The Cohen’s κ test was used to measure the level of agreement between reviewers [22]. Any disagreements about whether to include or exclude a study from the review were resolved through discussion among the authors until a consensus was reached.

### 2.5. Evidence Quality Assessment

A systematic assessment of the synthesis and quality of the evidence for each outcome was conducted in accordance with the Grading of Recommendations, Assessment, Development, and Evaluation (GRADE) approach [23]. The quality of the evidence was then categorized into four tiers: high, moderate, low, and very low. Due to the subjective nature of GRADE parameter evaluation, a separate evaluation was conducted by the three authors (M.P., W.N., and R.W.), with a consensus being reached through discussion and the use of Cohen’s k test.

### 2.6. Risk of Bias Across Studies

The procedural quality of each study included in this article was evaluated by two reviewers (M.M.P. and M.P.) that worked independently. The evaluation of study design, implementation, and analysis included the following criteria: the random allocation of study participants; inclusion/exclusion criteria that are clearly defined; and adequately balanced control/study groups within 10% of the participants; for a minimum double-blinded study, the calculated and necessary number of patients/pockets required for the study (power analysis); precisely defined severity of periodontitis; a precise method of obtaining results; and the application of statin gel in the study. One of the quality criteria assessments was whether the operator had undergone a calibration test before clinical measurements were taken. A score of 0–3 points indicated a high risk, 4–6 points denoted a moderate risk, and 7–9 points indicated a low risk. Discrepancies in scoring were resolved via discussion until a consensus was reached.

The scores for each study were calculated, and an overall estimated risk of bias (low, moderate, and high) was determined for each included study in accordance with the recommendations outlined in the Cochrane Handbook for Systematic Reviews of Interventions [24].

### 2.7. Data Extraction

Having reached a consensus regarding the selection of included articles, the two reviewers involved subsequently recorded the following data:

- Citation (first author and publication year);
- Type of study;
- Type of intervention/control group;
- Diagnosis;
- Clinical parameters evaluated;
- Outcomes;
- Length of follow-up;
- Number of participants;
- Whether a sample size calculation was performed;
- Age range and a standard deviation;
- Gender distribution;
- The country of research;
- Whether the research was carried out in a university center;

## 3. Results

### 3.1. Study Selection

**Figure 1:**
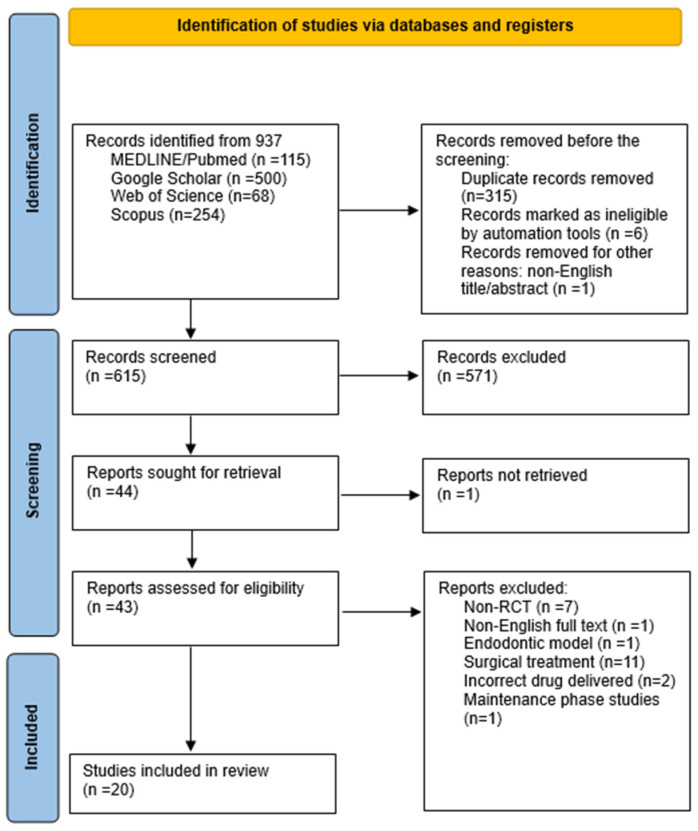
PRISMA 2020 flow diagram.

The research approach is illustrated in Figure 1 using a flowchart based on the PRISMA statement [21]. A primary search in the databases, with date filters applied, initially yielded 937 results. After removing duplicate studies, 615 studies underwent title and abstract screening. Subsequently, 43 studies underwent a full-text assessment, of which 20 articles met the eligibility criteria and were included in the review. All selected articles were randomized controlled trials (RCTs) published between 2012 and 2023.

Excluded studies that almost met the inclusion criteria included the use of statins in the surgical treatment of periodontitis [25,26,27,28,29,30,31,32,33,34,35] or non-RCT studies [36,37,38,39,40,41,42]. In addition, one study involved the administration of a statin in the form of a mouthwash [43], while another one involved the administration of a statinin the forms of an oral gel and a mouthwash [44]. One study concerned the maintenance phase [45] and another one was excluded due to the endodontic model [46]. One study was not available in English [47]. Table 3 presents the excluded studies and the reason for their extraction.

Out of the twenty articles, two were found to have a moderate risk of bias and eighteen to have a low risk. Eleven studies received the maximum number of points, with the two with the highest risk of bias receiving six out of nine. Five studies received eight points, while two studies received seven. No studies were eliminated due to low quality (high risk of bias) since the absence of information was considered unnecessary for ensuring this review’s thoroughness. If the response was affirmative, one point was given. On the other hand, a negative or ambiguous response did not receive any points. The bias risk assessment was divided into three categories: low, moderate, and high. Table 4 lists the results of the quality assessment and the risk of bias across the studies.

### 3.2. General Characteristics of the Included Studies

The number of study participants ranged from 20 to 104. Of the twenty studies, twelve were double-blinded, three were triple-masked, and three had a split-mouth design. Nineteen of the twenty studies determined the severity of periodontitis using the criteria of the previous periodontal disease classification [67]. One study used the criteria of the new classification [68]. One study involved patients diagnosed with aggressive periodontitis; eighteen studies covered patients with chronic periodontitis, while one study included patients with stage II periodontitis. The study population included patients with diabetes in three studies, smokers in three further studies, and generally healthy people in the remaining fourteen studies. One study specified postmenopausal women. Three studies did not report the distribution of the subjects by sex, while one study did not provide information about the age range of the participants, and eleven studies did not include information on the mean age and standard deviation. Three papers provided the mean age of the participants but did not include the standard deviation. Four studies included complete information on the distribution of subjects by sex, age range, and mean age. The collected data are categorized in Table 5.

All the studies presented evaluated the effect of the subgingival application of statins on periodontal clinical parameters as an adjunct to classical mechanotherapy in the non-surgical treatment of periodontal disease. Seventeen of the twenty studies also assessed the effect on radiological parameters, and one study assessed the effect on biochemical parameters.

In the studies presented, subgingival application involved 1.2% simvastatin gel [48,52,56,57,58,66], 1.2% gel with rosuvastatin [51,53,60], or 1.2% atorvastatin gel [52,54,59,63]. Three authors additionally compared the efficacy of two different statins with each other [49,50,62]. In three studies, the authors compared the efficacy of statins as an adjunct to non-surgical treatment with other chemicals administered subgingivally in gel form, i.e., 1% metformin [4], 2% melatonin [55], and 1% alendronate [65]. Follow-up periods ranged from 1 to 9 months. A summary of the key points from each article is presented in Table 6.

### 3.3. Main Study Outcomes

#### 3.3.1. Probing Pocket Depth (PPD)/Clinical Attachment Level (CAL)

In all presented studies, an inter-group comparison of clinical parameters PPD and relative CAL showed no statistically significant differences at the baseline. The statins group showed a significantly greater pocket depth reduction and relative CAL gain in various follow-ups compared to the control group with placebo gel. This trend also affected groups treated with other chemicals administered subgingivally [4,55,65] compared to groups with placebo gel. Pradeep et al. showed that the group recovering from anaddiction of the alendronate gel achieved statistically significant improvements in PD and CAL (*p* < 0.05) as compared to the group recovering from the addition of the atorvastatin gel in generally healthy patients [65]. In another study, Pradeep also showed that PD reduction and CAL gain post-treatment were significantly greater with the rosuvastatin gel than with the atorvastatin gel from baseline to the sixth month and from the sixth month to the ninth month [62]. Sharma et al. showed that in patients with diabetes mellitus, the rosuvastatin group had a significantly better improvement in RAL at 3 months and 6 months compared to the simvastatin group [49].

#### 3.3.2. Radiographic Parameters

In all studies that analyzed changes in radiological parameters (IBD, percentage of bone fill, RVAL, RHAL, and DDR), a statistically significant improvement was observed in the groups treated with the subgingival application of a statin compared to the group treated with the placebo gel. However, this was only evident at the six-month follow-up period. There was a similar trend in both generally healthy patients and patients with diabetes, as well as smokers. Sharma et al. showed that in patients with diabetes mellitus, the rosuvastatin group had a significantly better improvement in IBD at 6 months compared to the simvastatin group [49]. Panaj et al. showed that there was a significantly greater DDR% (*p*< 0.05) seen in the RSV group at the 6- and 12-month follow-up intervals as compared to the metformin group [4]. Pradeep et al. showed that DDR was significantly greater in the rosuvastatin group than in the atorvastatin group from baseline to the sixth month and from the sixth month to the ninth month post-treatment in generally healthy patients [62].

#### 3.3.3. Modified Sulcus Bleeding Index/Gingival Index (mSBI/GI)

An inter-group comparison of the mSBI/GI showed no statistically significant differences at the baseline in all presented studies, but there was a statistically significant decrease in this parameter in the statin group and the groups treated with other chemicals administered subgingivally [4,55,65] compared to the group with the placebo gel. This trend also affected smokers and diabetics [49,53,58,59,61,63]. Only one study found no statistically significant differences in mSBI between the baseline and after 6 months in the placebo group and statin group [51]. Moreover, Pradeep et al. showed that mSBI reduction between two statins from baseline to 6 months and from 6 to 9 months post-treatment was significantly greater with the rosuvastatin gel than the atorvastatin gel [62]. Martande et al. showed that a statistically significant decrease in mSBI was higher in the group with 1.2% ATV compared to the group with 1.2% SMV in the 3-, 6-, and 9-month follow-ups [50].

#### 3.3.4. Plaque Index (PI)

In fourteen of twenty studies, the authors found no statistically significant differences between full-mouth score or PI at the tested side in all groups during their study [4,48,50,51,52,53,57,58,59,61,63,64,65,66].

All studies demonstrated an acceptable tolerance of statins, with no side effects reported. Healing was uneventful.

#### 3.3.5. Osteocalcin (OC) Level

Moussa demonstrated that the osteocalcin level exhibited a notable decline exclusively in the groups treated with the simvastatin gel and the melatonin gel in conjunction with SRP, in comparison to the group treated with the locally administered placebo gel. Furthermore, a more pronounced reduction was observed in the group treated with 2% melatonin gel in comparison to the group treated with simvastatin gel [55].

#### 3.3.6. Anaerobic Colony Count

Chatterjee et al. showed no statistically significant difference in anaerobic bacterial colony counts between the SRP-treated group with the subgingival application of simvastatin and the SRP-treated group with placebo gel application after 6 months [51].

Additional details about the outcomes of the studies can be found in Table 6.

### 3.4. GRADE Rating

In order to reliably assess the individual parameters, the Grading of Recommendations Assessment, Development, and Evaluation (GRADE) tool was used [69,70]. All studies assessed PI and PPD parameters, with a total number of patients of 1192. The CAL index was tested on 919 patients in 16 studies, which was the least tested of the indices. Both mSBI/GI and radiographic parameters were assessed in 18 studies, with mSBI/GI assessed on a base of 1054 patients and radiographic parameters on a base of 1136 patients. In view of the inclusion of patients with diabetes and smokers, indirectness was assessed at a moderate level. All of the included studies were randomized trials, meaning they all began the assessment with a high level of evidence. Inconsistency was assessed in accordance with the GRADE handbook, with values of I^2^ < 40% indicating low heterogeneity, 30–60% indicating moderate heterogeneity, 50–90% indicating substantial heterogeneity, and 75–100% indicating considerable heterogeneity. The overall quality and summary of the evidence, as determined using the GRADE approach, are presented in Table 7.

## 4. Discussion

The objective of the systematic review presented here was to assess whether the application of statins subgingivally, in conjunction with conventional non-surgical periodontal disease treatment, offers additional benefits in clinical, radiological, and biochemical parameters in patients diagnosed with periodontal disease, including those who are generally healthy and those diagnosed with diabetes mellitus, as well as in patients who smoke.

The findings revealed a statistically significant improvement in PPD/CAL in the test group, where statin was used as an adjunct to the classic non-surgical treatment, in comparison to the placebo gel and SRP-treated group. It follows that the use of statins as an adjuvant to classical mechanotherapy may provide additional clinical benefits. This improvement was observed irrespective of the type of statin employed. It may be related to the therapeutic features of statins, including their capacity to regenerate, modulate the immune system, reduce inflammation, prevent thrombosis, inhibit microbial growth, and promote wound healing [49]. The improvement was more pronounced in patients without systematic diseases compared to those with type II diabetes [59] and in non-smokers compared to patients who smoke [63]. Greater improvements in clinical and radiological parameters occurred in patients diagnosed with aggressive periodontitis compared to patients with chronic periodontitis who were treated with both the local application of 1.2 SMV in combination with the SRP procedure [48].

In inter-group comparisons, statistically significant differences in improvements in PD and CAL clinical parameters were related to the type of statin used in the study. Pradeep indicated that PD reduction and CAL gain post-treatment were significantly greater with the 1.2% rosuvastatin gel than the 1.2% atorvastatin gel from baseline to the sixth month and from the sixth month to the 9thmonth in patients without any systematic diseases. The significance of clinical benefits with RSV over ATV might be explained by a greater anti-inflammatory action with a greater decrease in CRP levels with RSV than with ATV use [71,72]. RSV is a highly potent and efficacious hydrophilic statin as compared with other statins, and it is capable of inhibiting HMG-CoA reductase at lower pharmacological doses [53].

Sharma et al. showed that in patients with diabetes mellitus, the rosuvastatin group had a significantly better improvement in RAL at 3 months and 6 months compared to the simvastatin group. This may reflect the greater anti-inflammatory potential of rosuvastatin compared to simvastatin [49].

It is noteworthy that the observed differences in the efficacy of locally applied statins are consistent with the findings of studies examining the efficacy of systemic statins for the treatment of hypercholesterolemia [73]. Although the mechanism of action of all statins is similar, it seems that their potency depends on the type of statin.

An inter-group comparison of the mSBI/GI in all presented studies showed no statistically significant differences at the baseline. Still, there was a statistically significant decrease in this parameter with the statin group compared to the group with placebo gel in various follow-up periods. This trend also affected smokers and diabetics [49,53,58,59,61,63]. Only Chatterie et al. found no statistically significant differences in mSBI between the baseline and after 6 months when comparing the placebo group and the statin group [51].

Statins have been demonstrated to have anti-inflammatory effects by inhibiting GTPases, reducing the production of IL-6 and IL-8 in human oral epithelial cells, lowering the cluster of differentiation thirty-six expression, and lowering NF-KB levels. Statins also inhibit the secretion of MMP-1, MMP-2, and MMP-9 and have been shown to diminish AGE-induced intracellular ROS [74], which has the effect of reducing mSBI. In summary, significantly greater improvements in mSBI, PD, and CAL in the statin groups compared with the placebo groups are most likely due to their anti-inflammatory, antioxidant, immunomodulatory, and endothelium-stabilizing actions [75].

In all studies that analyzed changes in radiological parameters, a statistically significant improvement was observed in the groups treated with the subgingival application of a statin compared to the group treated with the placebo gel. However, this was only evident after a six-month follow-up period. Significant advantages over the placebo in radiographic parameters can be ascribed to statins’ ability to promote angiogenesis, inhibit bone resorption via the upregulation of osteoprotegerin [75], and increase osteoblastic differentiation by stimulating the expression of bone morphogenic protein 2, thus inducing bone formation [76]. Statins protect the alveolar bone by suppressing osteoclast development by inhibiting MMPs and RANKL expression and also by increasing BMP-2 and VEGF in human PDL cells, which helps them proliferate and differentiate into osteoblasts [62].

Sharma et al. showed that in patients with diabetes mellitus, the rosuvastatin group had a significantly better improvement in IBD at 6 months compared to the simvastatin group [49]. Panaj et al. showed that there was significantly greater DDR% (*p*< 0.05) seen in the RSV group at the 6- and 12-month intervals as compared to the metformin group [4]. Pradeep et al. showed that DDR post-treatment was significantly greater in the rosuvastatin group than in the atorvastatin group from baseline to the sixth month and from the sixth month to the ninth month in generally healthy patients. The findings substantiate the assertion that statins exert pleiotropic effects on both hard and soft tissues; however, the intensity of these effects may vary and is contingent on the specific statin employed.

One important non collagenous component of bone is osteocalcin [69]. Osteoblasts, odontoblasts, and chondrocytes are the main cells that synthesize and secrete it [70]. In periodontitis, where bone hemostasis is disrupted by a higher resorption rate, OC is thought to be a bone formation biomarker and plays a significant role in bone remodeling. However, in this condition, it attracts osteoclasts to the site of bone degradation by encouraging their differentiation into active osteoclasts. As a result, OC is now widely acknowledged as a marker of bone turnover as opposed to bone formation [77]. Conclusively, osteocalcin could be used as a prognostic marker to predict the probable outcome of the disease [78]. Variable osteocalcin GCF levels between healthy and disease states may highlight the abnormal bone turnover occurring in periodontitis, and the reduction in osteocalcin levels as a result of treatment may indicate a normalization of bone metabolism.

Chatterjee et al. showed no statistically significant difference in anaerobic bacterial colony counts between the SRP-treated group with the subgingival application of simvastatin and the SRP-treated group with placebo gel application after 6 months [51]. This result may demonstrate the positive impact on the ecosystem of the SRP procedure, which is the gold standard in the treatment of periodontal disease.

In fourteen of twenty studies, the authors found no statistically significant differences between full-mouth score or PI at the tested side in all groups during their study compared to baseline [4,48,50,51,52,53,57,58,59,61,63,64,65,66]. This indicates that optimal oral hygiene was maintained throughout the study period. Six studies [49,54,55,56,60,62] reported a statistically significant improvement in PI compared to baseline; this can be explained by improved plaque control inpatients through the continual motivation and reinforcement of oral hygiene recommendations.

According to recent research, people with periodontitis have a reduced ability of the oral microbiota to reduce nitrates [79]. Through a number of mechanisms and pathways, statins have been shown to upregulate endothelial NO synthase (eNOS), increasing NO bioavailability. They also improve eNOS expression by prolonging the half-life of eNOS mRNA. This is accomplished by localizing the eNOS mRNA, altering the cytoskeleton, and inhibiting RhoA geranyl-geranylation. Moreover, it has been shown that statins decrease caveolin-1, a crucial membrane protein that binds eNOS and prevents it from producing NO. Data indicate that statin therapy may activate the pathway of phosphatidylinositol 3-kinase (PI3K)/protein kinase Akt, which raises eNOS and controls cellular growth, survival, and proliferation. It has been shown that statins inhibit the expression of activator protein-1, hypoxia-inducible factor-1alpha, and nuclear factor kappa B (NF-kappaB) in endothelial cells. Later on, these elements influence pro-inflammatory cytokines, chemokines, adhesion molecules, and growth factors. Additionally, statins may affect the phenotype of T cells by promoting the expression of FoxP3 regulatory T cells, which triggers immune tolerance, and by modulating the differentiation of pro-inflammatory IL-17 helper T cells. Major histocompatibility complex II is expressed by antigen-presenting cells and is multiplied in the presence of interferon-γ. It has been shown that statins decrease the expression of major histocompatibility complex II on antigen-presenting cells, which in turn decreases T-cell activation. Additionally, statins have the ability to attach to an allosteric site in the β2-integrin function-associated antigen-1 protein, which inhibit it and reduce lymphocyte adhesion as well as T-cell co-stimulation [15].

Diabetes mellitus (DM) and chronic periodontitis have a long-standing bidirectional association [49]. Diabetics experience increased frequency and intensity of periodontal disease [80,81]. The sixth DM complication has been named as periodontitis. Hyperglycemia causes the emergence of elevated levels of advanced glycation-end products (AGEs), such as tumor necrosis factor (TNF) and reactive oxygen species (ROS). These substances have the potential to impact osteoclast production and reduce collagen synthesis in osteoblasts [82].

In addition, patients with DM have a reduced chemotactic and phagocytic capacity of multinucleated phagocytes. The inflammatory response to bacterial plaque build-up usually occurs earlier and is usually more severe. Monocytes produce more pro-inflammatory cytokines in patients with T1D than in patients without diabetes. These elevated levels can be measured in the gingival crevicular fluid [79].

Periodontal therapy significantly improves glycemic control [83,84]. Studies show that the topical application of antimicrobial drugs in patients with DM can help reduce TNF-alpha level [85,86]. There are studies suggesting that the addition of a statin locally in addition to classic non-surgical therapy may provide additional clinical benefits, so this step is worth considering in patients in this reference group [49,59,61].

It is a well-documented fact that smoking is the most important environmental risk factor of periodontitis, and it is one of the significant risk indicators of attachment loss, and smokers tend to have greater numbers of deeper periodontal pockets than non-smokers [87,88,89]. Patients who smoke achieve worse outcomes with non-surgical periodontal treatment compared to non-smoking patients. Moreover, PD reduction and CAL gain achieved are also lower in smokers after periodontal surgery [90,91].

In the case of smokers, there are studies suggesting defects in neutrophil function, impaired serum antibody responses to periodontal pathogens, and potentially diminished gingival fibroblast function [92,93]. This impairment of immune function translates into a poorer response to periodontal treatment. There are indications that the addition of a statin subgingivally in addition to classical non-surgical therapy may improve the results achieved in smokers [53,58,63], so it seems worth considering the use of subgingival statins in this reference group to support classical periodontal therapy.

Although this was not the subject of the above systematic review, similar trends in improved clinical and radiological parameters were observed in studies where statins were incorporated into surgical treatment protocols [25,26,27,28,29,30,31,32,33,34,35]. These studies employed statins as the sole adjuvant or in combination with platelet-rich fibrin [27,28,31,32,33,34,35,94]. Another study demonstrated the efficacy of a combination therapy comprising SRP, statins, and photodynamic therapy when compared to the SRP-alone procedure [42]. These studies highlight the multifaceted potential of statins in the management of periodontal disease.

The advent of localized drug delivery systems makes it possible to achieve higher concentrations of the active substance directly in the gingival pocket, thus avoiding adverse systemic effects, facilitating the acquisition of patient consent and cooperation, and potentially improving treatment outcomes. It is also worth noting the results of the study groups that were treated with the SRP procedure in combination with substances other than statins (melatonin gel, metformin gel, and alendronate gel) [4,55,65]. Although further studies are needed to assess their efficacy, they indicate the potential that local drug delivery has in the treatment of periodontal disease.

The current systematic review and the studies discussed in it have some limitations:

1. The systematic review is narrative and does not include statistical tests;
2. The methodology used to assess clinical parameters, using a probe with calibration in 1 mm increments, carries a risk of measurement reading error, although the use of an acrylic stent and the performance of an operator calibration test appear to minimize this risk;
3. Six studies found a statistically significant difference between baseline PI levels and subsequent PI measurements, making it difficult to conclude whether the improvement in clinical parameters was due to the additional effect of the statin or due to improved oral hygiene;
4. In none of the presented studies, the statin concentration was assessed by an external entity, which would be an objective confirmation of the drug’s concentration during preparation;
5. Three studies did not use double-blinding, which may have affected the study results presented, and only three authors designed a split-mouth study;
6. Only three studies were completed after 2020, highlighting the need for more up-to-date studies on this topic;
7. Nineteen of the twenty papers were conducted in India, so the conclusions may relate to a narrow population;
8. The authors used different indices and different clinical/radiological parameters, making it difficult to compare their results;
9. A group of diabetics and smokers, with additional factors that may modify the course of periodontal disease, and their response to treatment were included in the analysis, which may affect the final results;
10. Most of the work focused on the change in clinical and radiological parameters under the influence of statins; there is a need for more up-to-date studies with careful methodology, focusing on biological, biochemical, and histological aspects with a more representative study group to definitively confirm their efficacy as an adjunct to classical mechanotherapy;
11. Of the twenty included articles, seven were published by Pradeep et al., which generates the risk of patient outcomes duplication between studies. Nevertheless, the studies from Pradeep et al. were generated within 7 years on different study groups of patients, with/without diabetes and with different degrees of periodontitis. The mean age of patients also varied within the studies. The authors found no indication that the results of the same patients were replicated in multiple studies from the same authors.

Long-term, multi-center, randomized, controlled clinical trials are needed to confirm the clinical, biological, biochemical, and histological effects of statins on periodontal parameters. These studies should help determine the optimal vehicle, dose, and type of statin to be used depending on the clinical situation. The resulting data will allow for the development of clinical protocols for the treatment of periodontal disease, which include the use of subgingival statins and may be particularly important in patients with general diseases who have impaired tissue metabolism associated with the underlying disease.

## 5. Conclusions

The preliminary evidence suggests that the subgingival application of statins in combination with SRP may result in additional clinical and radiographic improvements in patients diagnosed with periodontal disease. The potential benefits of this approach may be particularly relevant for individuals with periodontal disease who are also diabetic or smokers.

## Figures and Tables

**Table 1:** Syntaxes used, limitations and number of results for each of the databases searched.

Database	Search Terms	Limitations	Results
PubMed	Periodontitis [Mesh] AND Statin [Mesh] OR Simvastatin [Mesh] OR Atorvastatin [Mesh] or Rosuvastatin Calcium [Mesh]	Years 2012–2024 English language	115
Scopus	KEY (periodontitis) OR KEY (periodontal AND disease) OR KEY (periodontal AND therapy) AND KEY (statin) OR KEY (hydroxymethylglutaryl-coa AND reductase AND inhibitors) OR KEY (simvastatin) OR KEY (rosuvastatin) OR KEY (atorvastatin)	Years 2012–2024 English language	254
Web of Science	AK = (Periodontitis OR Periodontal AND disease OR Periodontal AND therapy) AND AK = (Statin OR Hydroxymethylglutaryl-coA AND Reductase AND Inhibitors OR Simvastatin OR Rosuvastatin OR Atorvastatin)	Years 2012–2024 English language	68
Google Scholar	Periodontitis” OR “Periodontal disease” OR “Periodontal therapy” AND “Statin” OR “Hydroxymethylglutaryl-ciA Reductase Inhibitors” OR “Simvastatin” OR “Rosuvastatin” OR „Atorvastatin”	Years 2012–2024 English language Search limited to 500 most accurate results	500

**Table 2:** Selection criteria for papers included in this systematic review.

Inclusion Criteria	Exclusion Criteria
Full-text available English language Adult patients with periodontitis Randomized control clinical trial (RCT) Compared SRP+ subgingival statin gel vs. SRP+ placebo or SRP alone Reported results in terms of clinical parameters PPD CAL changes after at least 3 months The intervention group should use a statinas the sole adjunct to non-surgical mechanical periodontal treatment Patients with diabetes mellitus and smokers with periodontitis were included in this analysis	Nonrandomized trials Case reports/case series reviews Systematic reviews Meta-analysis Historic reviews Conferencepapers Letters to editors Animal studies Patients younger than 18 years old Studies without mechanical periodontal therapy Patients during the maintenance phase Statins used with any other drug/biomaterial in the same study group Patients receiving statins systemically Studies published before 2012

**Table 3:** A list of excluded studies and the main rationale for their exclusion.

Reason for Exclusion	Reference Number
Non-RCT	[36,37,38,39,40,41,42]
Surgical treatment	[25,26,27,28,29,30,31,32,33,34,35]
Maintenance phase	[45]
Non-English full text	[47]
Endodontic model	[46]
Incorrect drug delivered	[43,44]

**Table 4:** The results of the quality assessment and the risk of bias across the studies.

Criteria	Prianka et al. (2017) [48]	Sharma et al. (2023) [49]	Martande et al. (2016) [50]	Chatterie et al. (2019) [51]	Pradeep et al. (2012) [52]	Kanoriya et al. (2019) [53]	Shirke et al. (2019) [54]	Moussa (2021) [55]	Gujari et al. (2015) [56]	Gupta et al. (2021) [57]	Rao et al. (2013) [58]	Kumari et al. (2016) [59]	Predeep et al. (2015) [60]	Predeep et al. (2012) [61]	Pankaj et al. (2018) [4]	Predeep et al. (2016) [62]	Kumari et al. (2016) [63]	Predeep et al. (2013) [64]	Predeep et al. (2016) [65]	Predeep et al. (2010) [66]
Random allocation	1	1	1	1	1	1	1	1	0	1	1	1	1	1	1	1	1	1	1	1
Inclusion/exclusion criteria clearly defined	1	1	1	1	1	1	1	1	1	1	1	1	1	1	1	1	1	1	1	1
Operator calibration test conducted	1	1	1	0	1	1	1	0	1	0	1	1	0	1	1	1	1	1	1	0
Balanced study groups	1	1	1	1	1	1	1	1	1	1	1	1	1	1	1	1	1	1	1	1
Minimum double-blinded study	1	1	0	1	0	1	0	1	1	0	1	1	1	1	1	1	1	1	1	0
Calculated study group (power analysis)	1	1	1	1	1	1	1	0	0	0	1	1	1	1	1	1	1	1	1	1
Precisely defined severity of periodontitis	1	1	1	1	1	1	1	1	1	1	1	1	1	1	1	1	1	1	1	1
Clear method of obtaining statin gel	1	1	1	1	1	1	1	1	1	1	1	1	1	1	1	1	1	1	1	1
Well-defined method of application	1	1	1	1	1	1	1	0	1	1	1	1	1	1	1	1	1	1	1	1
Total	9	9	8	8	8	9	8	6	7	6	9	9	8	9	9	9	9	9	9	7
Risk of bias	LOW	LOW	LOW	LOW	LOW	LOW	LOW	MDR	LOW	MDR	LOW	LOW	LOW	LOW	LOW	LOW	LOW	LOW	LOW	LOW

Abbreviation: MDR—moderate.

**Table 5:** Characteristics of patients by study.

Author/Year	Country	Setting	Study Design	Sample Size Calculation	Study Population	Patients	Sex		Mean Age (Years) (±SD)	Age Range (Years)
							**Female**	**Male**		
Priyanka et al. (2017) [48]	India	University	RCT, double-blinded	Yes	Patients with aggressive periodontitis	24	10	14	no data	30–50
Sharma et al. (2023) [49]	India	University	RCT, double-blinded	Yes	Patients with moderate chronic periodontitis and type II diabetes mellitus	30	no data	no data	45.70 (±5.31)	35–60
Martande et al. (2017) [50]	India	University	RCT	Yes	Patients with moderate to severe chronic periodontitis	96	46	50	no data	30–50
Chatterjee et al. (2019) [51]	India	University	RCT, double-blinded	Yes	Patients with chronic periodontitis	100	53	47	no data	30–60
Pradeep et al. (2012) [52]	India	University	RCT	Yes	Patients with chronic periodontitis and buccal class II furcation defects	72	34	38	no data	30–50
Kannoriya et al. (2019) [53]	India	University	RCT, triple-masked	Yes	Patients and smokers with chronic periodontitis	60	0	60	no data	30–50
Shirke et al. (2019) [54]	India	University	RCT, split-mouth	Yes	Patients with chronic periodontitis and a minimum of one pair of bilateral intraosseous defects	20	10	10	35.7 (no data)	30–45
Moussa (2021) [55]	Egypt	University	RCT, double-blinded	No	Patients with stage II periodontitis	36	23	13	41.25 (±8.84)	21–55
Gujari et al. (2015) [56]	India	University	RCT, double-blinded, split-mouth	No	Postmenopausal women with moderate-to-severe periodontitis with contralateral periodontal pockets	20	20	0	50 (±5)	45–55
Gupta et al. (2021) [57]	India	University	RCT, split-mouth	No	Patients with chronic periodontitis	25	no data	no data	Group I: 32.50 (±8.182) Group II: 32.50 (±8.121)	no data no data
Rao et al. (2013) [58]	India	University	RCT, double-blinded	Yes	Patients and smokers with chronic periodontitis	40	0	40	no data	30–50
Kumari et al. (2016) [59]	India	University	RCT, double-blinded	Yes	Patients with chronic periodontitis and type II diabetes mellitus	75	37	38	45.5 (no data)	40–50
Pradeep et al. (2015) [60]	India	University	RCT, double-blinded	Yes	Patients with moderate chronic periodontitis	70	37	33	no data	25–55
Pradeep et al. (2012) [61]	India	University	RCT, double-blinded	Yes	Patients with chronic periodontitis and type II diabetes mellitus	38	18	20	no data	30–50
Pankaj et al. (2018) [4]	India	University	RCT, triple-masked	Yes	Patients with moderate-to-advanced chronic periodontitis	90	46	44	34.32 (± 5.09)	25–45
Pradeep et al. (2016) [62]	India	University	RCT, triple-masked	Yes	Patients with chronic periodontitis	90	45	45	36 (no data)	25–45
Kumiari et al. (2016) [63]	India	University	RCT, double-blinded	Yes	Patients and smokers with chronic periodontitis	71	no data	no data	no data	30–50
Pradeep et al. (2012) [64]	India	University	RCT, double-blinded	Yes	Patients with severe chronic periodontitis	67	32	35	no data	30–50
Pradeep et al. (2016) [65]	India	University	RCT, double-blinded	Yes	Patients with chronic generalized periodontitis	104	51	53	no data	30–50
Pradeep et al. (2019) [66]	India	University	RCT	Yes	Patients with moderate chronic periodontitis	64	31	33	30.5 (±4.1)	25–45

Abbreviation: SD—standard deviation and RCT—randomized controlled trial.

**Table 6:** Detailed characteristics of the studies included in this review.

Author/Year	Study Population (Population)	Test Group (Intervention)	Control Group (Comparison)	Results (Outcome)	Follow-Up Period
Non-Smoking Patients without Diabetes					
Priyanka et al. (2017) [48]	Patients with aggressive periodontitis	1.SRP+ 1.2 mg SMV gel	2.SRP+placebo gel Parameters evaluated: PI, mSBI, PD, CAL, and IBD	No side effects. No difference in PI between groups. A decline in mSBI in both groups. Group 1 with a larger decline after six months. In groups 1 and 2, the mean PD decreased at 6 months and was 1.14 ± 0.04 mm and 3.78 ± 0.62 mm, respectively. Bone fill in the statins group increased.	3–6 months
Martande et al. (2016) [50]	Patients with moderate-to-severe chronic periodontitis	1.SRP+1.2% ATV gel 2.SRP+1.2% SMV gel	3.SRP+placebo gel Parameters evaluated: PI, mSBI, PD, RAL, and IBD	No side effects. No difference in PI between groups. A decrease in the ATV group’s mSBI values compared to the SMV group. Both groups with PD hada reduction and an RAL gain. ATV group displayed higher mean PD reduction and mean RAL gain. ATV group had a higher proportion of reduced radiographic defect depth.	3–6–9 months
Chatterjee et al. (2019) [51]	Patients with chronic periodontitis	1.SRP+1.2% RSV gel	2.SRP+placebo gel Parameters evaluated: PI, mSBI, PD, CAL, IBD, and anaerobic colony count	No side effects. Comparable mSBI scores between groups. Periodontal parameterimprovements in both groups. Group 1 had an increase in CAL. The PD in the statin group decreased. Bone fill in the statins group increased. No differences between groups for mSBI, PI, or anaerobic colony count.	6 months
Pradeep et al. (2012) [52]	Patients with chronic periodontitis and buccal class II furcation defects	1.SRP+ 1.2 mg SMV gel	2.SRP+placebo gel Parameters evaluated: PI, mSBI, PD, RVAL, RHAL, and bone fill	No side effects. No difference in PI between groups. Statins group mSBI score decreased. Mean RVAL and RHAL increased in statins group. Bone fill in the statins group increased.	3–6 months
Shirke et al. (2019) [54]	Patients with chronic periodontitis and a minimum of one pair of bilateral intraosseous defects	1.SRP+1.2% ATV gel	2.SRP+placebo gel Parameters evaluated: PI, mSBI, PPD, CAL, and bone fill	Acceptable statin toleration and no side effects. Significant reduction in the PI, mSBI, and PPD at 3 and 6 months in both groups. Higher increase in CAL in the ATV gel group after 3 and 6 months. After 6 months, the group with ATV gel had a considerably higher bone fill.	3–6 months
Moussa (2021) [55]	Patients with stage II periodontitis	1.SRP+1.2% ATV gel 2.SRP+2% melatonin gel	3.SRP+placebo gel Parameters evaluated: PI, GI, PPD, CAL, and OCT	No side effects. Regarding PI and GI, there was a reduction in all groups. PI and GI reduction was more significant in ATV gel group and melatonin gel group. Greater improvements in PD and CAL in the ATV gel and melatonin gel groups. Greater reductions in the osteocalcin level in the melatonin gel group compared with the ATV gel group; no reduction in the osteocalcin level in the placebo gel group.	3 months
Gaekwad et al. (2015) [56]	Postmenopausal women with moderate-to-severe periodontitis with contralateral periodontal pockets	1.SRP+1.2% SMV gel	2. SRP+placebo Parameters evaluated: PI, GI, BI, PPD, and CAL	Acceptable statin toleration and no side effects. Significant decreases in PI, GI, BI, and PPD and asignificant increase in CAL in both groups at 6 months; SMV gel group had a greater reduction inPI, GI, BI, and PPD and abetter gain in CAL compared to placebo gel group at 6 months.	3–6 months
Gupta et al. (2021) [57]	Patients with chronic periodontitis	1.SRP+1.2% SMV gel	2.SRP+placebo gel Parameters evaluated: PI, mSBI, PPD, CAL, and RAL	Acceptable statin toleration and no side effects. No significant difference in PI between groups in any visit. The SMV group’s mSBI was significantly lower than that of the placebo group after 3 and 6 months. After 3 and 6 months, a greater increase in CAL and a reduction in PD were seen in SMV gel group compared to placebo gel group.	3–6 months
Pradeep et al. (2015) [60]	Patients with moderate chronic periodontitis	1.SRP+1.2% RSV gel	2.SRP+placebo gel Parameters evaluated: PI, mSBI, PD, CAL, and IBD	No side effects. An improvement in PI after 1 and 4 months in both groups. A greater decrease in mSBI scores in the group with RSV. The mean decrease in PD after 6 months was greater in the RSV gel group. Mean CAL gain and mean IBD after 6 months were higher in the RSV gel group.	1–3–4–6 months
Pankaj et al. (2018) [4]	Patients with moderate-to-advanced chronic periodontitis	1.SRP+1.2% RSV gel 2.SRP+1% MF gel	3.SRP+placebo gel Parameters evaluated: PI, mSBI, PD, CAL, IBD, and DDR%	No side effects. No difference in PI between groups. A decrease in mSBI in all three groups. PD and CAL values in the SMV and MF groups were lower The reductions in IBD depth and DDR% in the test groups were noticeably higher. PD and CAL values in the test groups were lower than in the placebo group. DDR% was higher in the RSV group than in the MF group.	6–12 months
Pradeep et al. (2016) [62]	Patients with chronic periodontitis	1.SRP+1.2% RSV gel 2.SRP+1.2% ATV gel	3.SRP+placebo gel Parameters evaluated: PI, mSBI, PD, CAL, and IBD depth	No side effects. In all groups, an increase in CAL and a decrease in PD, mSBI, and PI were seen. In the statin groups, DDR% was significant. Between the two statins, there was no difference in the mean PI and mSBI reductions. Compared to ATV gel group, RSV gel group demonstrated higher PI, PD reduction, CAL gain, and DDR.	6–9 months
Pradeep et al.(2012) [64]	Patients with severe chronic periodontitis	1.SRP+1.2% ATV gel	2.SRP+placebo gel Parameters evaluated: PI, PD, CAL, and IBD	No side effects. No difference in PI between groups. The test group’s mSBi significantly dropped. Higher PD reduction and CAL gain in the ATV group. IBD demonstrated a significant mean reduction in the ATV group. Worse vertical radiographic defect fill in the ATV group.	3–6–9 months
Pradeep et al. (2016) [65]	Patients with chronic generalized periodontitis	1.SRP+1% ALN gel 2.SRP+1.2% ATV gel	3.SRP+placebo gel Parameters evaluated: PI, SBI, PD, CAL, IBD, and DDR%	No side effects. No difference in PI between groups. Decrease in mSBI and a greater reduction in PD, CAL, and IBD as well as a significantly higher DDR% in the test groups. Greater improvements in PD, CAL, and DDR% in the ALN group compared to the ATV group.	3–6–9 months
Pradeep et al. (2009) [66]	Patients with moderate chronic periodontitis	1.SRP+ 1.2 mg SMV gel	2.SRP+placebo gel Parameters evaluated: PI, mSBI, PD, CAL, IBD, and concentration of SMV in GCF	No side effects. No difference in PI between groups. The mSBI scores decreased in both groups. The mSBI score decreased more in the test group. Decrease in PD in both groups. CAL increment was higher in the test group. Mean IBD in the simvastatin group decreased more than in the placebo gel group. Two hours after application, SMV in GCF reached its peak.	1–2–4–6 months
Diabetic Patients					
Kumari et al. (2016) [59]	Patients with chronic periodontitis and type II diabetes mellitus	1.SRP+1.2% ATV gel	2.SRP+placebo gel Parameters evaluated: PI, mSBI, PD, RAL, and IBD	Acceptable statin toleration and no side effects. No difference in PI between groups. The ATV gel group had a higher reduction in mSBI, PD, and IBDs and a greater increase in RAL compared to the placebo gel group.	3–6–9 months
Pradeep et al. (2012) [61]	Patients with chronic periodontitis and type II diabetes mellitus	1.SRP+1.2% SMV gel	2.SRP+placebo gel Parameters evaluated: PI, mSBI, PD, CAL, and IBD	Acceptable statin toleration and no side effects. No difference in PI between groups. A decrease in mSBI, a reduction in IBD and PD, a greater increase in CAL, and a greater filling of vertical cavities in test group.	3–6–9 months
Sharma et al. (2023) [49]	Patients with moderate chronic periodontitis and type II diabetes mellitus	1.SRP+1.2% SMV gel 2.SRP+1.2% RSV gel	3.SRP+placebo gel Parameters evaluated: PI, mSBI, PPD, RAL, and IBD	No side effects. Improvements were observed in all groups for PI, mSBI, PPD, RAL, and IBD. Control group showednon-significant improvements in RAL and IBD but significantly greater improvements in PI, mSBI, and PPD. Test groups had improvements in PI, mSBI, IBD, PPD, and RAL. RSV group had improvements in IBD and RAL compared to SMV group.	3–6 months
Smoking Patients					
Kumiari et al. (2016) [63]	Patients and smokers with chronic periodontitis	1.SRP+1.2% ATV gel	2.SRP+placebo gel Parameters evaluated: PI, PD, CAL, and IBD	Acceptable statin toleration and no side effects. No difference in PI between groups. mSBI was significantly lower in the ATV group. The ATV group had a higher PD, IBD reduction, and CAL gain.	3–6–9 months
Rao et al. (2012) [58]	Patients and smokers with chronic periodontitis	1.SRP+1.2% SMV gel	2.SRP+placebo gel Parameters evaluated: PI, mSBI, PD, CAL, and IBD	Acceptable statin toleration and no side effects. No difference in PI between groups. Decreases in mSBI and IBD, a greater PD reduction, and CAL gain was seen in the SMV group. A greater vertical cavity filling in the SMV group.	3–6–9 months
Kannoriya et al. (2019) [53]	Patients and smokers with chronic periodontitis	1.SRP+1.2% RSV gel	2.SRP+placebo Parameters evaluated: PI, mSBI, PD, CAL, and IBD	Acceptable statin toleration and no side effects. No significant difference in PI between groups. The RSV group had greater improvements in mSBI, CAL, PD, and IBD.	3–6–9 months

Abbreviations: SRP—scaling root planning, SMV—simvastatin, PI—plaque index, mSBI—modified sulcus bleeding index, PD—pocket depth index, CAL—clinical attachment level, IBD—intrabonydefect depth, RSV—rosuvastatin, PPD—probing pocket depth, RAL—relative attachment level, ATV—atorvastatin, RVAL—relative vertical attachment, RHAL—relative horizontal attachment level, OCT—osteocalcin GI—gingival index, BI—bleeding index, MF—metformin, DDR%—defect depth reduction (%), ALN—alendronate, and GCF—gingival crevicular fluid.

**Table 7:** Summary of findings (SoFs) and quality of evidence (GRADE).

Outcome	Number of Studies	Number of Patients	Study Design	Risk of Bias	Inconsistency	Indirectness	Imprecision	Publication Bias	Quality of Evidence	Importance
PPD	20	1192	RCT	Low	Low	Moderate	Low	No detected	High	Critical
CAL	16	919	RCT	Low	Low	Moderate	Low	No detected	High	Critical
PI	20	1192	RCT	Low	Low	Moderate	Low	No detected	High	Important
mSBI/GI	18	1054	RCT	Low	Low	Moderate	Low	No detected	High	Important
Radiographic parameters	18	1136	RCT	Low	Low	Moderate	Low	No detected	High	Critical

RCT—randomized controlled trial.

## Data Availability

No new data were created or analyzed in this study.
